# Phytoremediation of pollutants in oil-contaminated soils by *Alhagi camelorum*: evaluation and modeling

**DOI:** 10.1038/s41598-024-56214-y

**Published:** 2024-03-06

**Authors:** Bahador Nemati, Mohammad Mehdi Baneshi, Hossein Akbari, Rouhullah Dehghani, Gholamreza Mostafaii

**Affiliations:** 1 Department of Environmental Health Engineering, School of Health, Kashan University of Medical Sciences, Kashan, Iran; 2 Social Determinants of Health Research Center, Yasuj University of Medical Sciences, Yasuj, Iran; 3 Department of Biostatistics and Epidemiology, School of Health, Kashan University of Medical Sciences, Kashan, Iran; 4 Social Determinants of Health (SDH) Research Center, and Department of Environment Health, Kashan University of Medical Sciences, Kashan, Iran

**Keywords:** Phytoremediation, Total petroleum hydrocarbons, Heavy Metals, *Alhagi camelorum*, Kinetic Rate, Biotechnology, Environmental sciences

## Abstract

Phytoremediation is a cost-effective and environmentally friendly method, offering a suitable alternative to chemical and physical approaches for the removal of pollutants from soil. This research explored the phytoremediation potential of *Alhagi camelorum*, a plant species, for total petroleum hydrocarbons (TPHs) and heavy metals (HMs), specifically lead (Pb), chromium (Cr), nickel (Ni), and cadmium (Cd), in oil-contaminated soil. A field-scale study spanning six months was conducted, involving the cultivation of *A. camelorum* seeds in a nursery and subsequent transplantation of seedlings onto prepared soil plots. Control plots, devoid of any plants, were also incorporated for comparison. Soil samples were analyzed throughout the study period using inductively coupled plasma-optical emission spectroscopy (ICP‒OES) for HMs and gas chromatography‒mass spectrometry (GC‒MS) for TPHs. The results showed that after six months, the average removal percentage was 53.6 ± 2.8% for TPHs and varying percentages observed for the HMs (Pb: 50 ± 2.1%, Cr: 47.6 ± 2.5%, Ni: 48.1 ± 1.6%, and Cd: 45.4 ± 3.5%). The upward trajectory in the population of heterotrophic bacteria and the level of microbial respiration, in contrast to the control plots, suggests that the presence of the plant plays a significant role in promoting soil microbial growth (*P* < 0.05). Moreover, kinetic rate models were examined to assess the rate of pollutant removal. The coefficient of determination consistently aligned with the first-order kinetic rate model for all the mentioned pollutants (R^2^ > 0.8). These results collectively suggest that phytoremediation employing *A. camelorum* can effectively reduce pollutants in oil-contaminated soils.

## Introduction

From a worldwide perspective, soil is recognized as the third vital component of the environment, after water and air. The presence of healthy soil is essential for life on Earth, as 95% of the food consumed by humans is derived from the soil. Planning for the preservation of healthy soil is crucial to ensure human survival. The introduction of pollutants into the soil leads to changes in soil quality and gives rise to environmental issues^1^. Environmental pollution, stemming from human activities and the release of pollutants, has given rise to a range of environmental issues. One notable problem among these is the contamination of soil with petroleum compounds in regions abundant in oil and in the vicinity of oil refineries^2^. Crude oil stands as one of the paramount energy sources globally, yet its extensive production, transportation, consumption, and disposal processes contribute significantly to large-scale environmental pollution^3^.

In countries with ongoing exploration activities, facilities, refineries, and abundant oil resources, the leakage, seepage, and infiltration of petroleum pollutants and derivatives into the soil are regarded as some of the most substantial soil contaminants^4^. In these regions, there exists the potential for a substantial annual influx of hazardous pollutants into the environment, which may result in adverse effects on the ecosystem^5^. Factors such as drilling, the emission of pollutants from refineries and power plants, leakage from oil reservoirs, tanker accidents, and oil spills all contribute to the escalation of pollution issues in the surrounding soils of these areas^6^.

One category of pollutants introduced into the soil through crude oil comprise HMs^7^. HMs stand out as the most significant soil pollutants, known for their adverse effects on human health and their potential to diminish both agricultural production and product quality^4^. While these metals naturally occur in soil in trace amounts and are deemed essential nutrients for plants in small quantities, elevated levels can lead to toxicity, posing hazards to both plant life and human well-being^8^. HMs are biologically nondegradable, and owing to their extended half-life, they can exert long-term detrimental effects on the soil and its biological processes. Moreover, they have the potential to induce various diseases in humans. The nondegradability of HMs has positioned them as one of the most hazardous groups of environmental pollutants^2^.

These Mineral salts can infiltrate aquatic ecosystems via wastewater discharge, sewage, rainfall runoff, and atmospheric deposition^4^. HMs exhibit a pronounced inclination to amass within the bodies of aquatic organisms, leading to their accumulation^7^. By consuming these organisms, pollution permeates higher trophic levels and ultimately reaches humans, positioned at the apex of the food chain, posing a threat to their health^9^. The presence of HMs in environmental pollution can lead to various health repercussions, including strokes and heart attacks, cancers, heightened kidney issues, genetic disorders, mental and psychological disorders, the birth of infants with defects, diminished intelligence quotient, and a lack of concentration^10^.

Another pollutant that infiltrates the soil through crude oil is petroleum hydrocarbon. Petroleum hydrocarbons are the most significant sources of environmental pollution on a global scale^11^. Soil pollution with petroleum hydrocarbons is a major concern in areas involved in oil extraction. In recent decades, there has been significant attention given to the environmental impact of hydrocarbon pollution^5^. The existence of petroleum hydrocarbons in soil can adversely affect biological processes conducted by soil microorganisms, leading to toxicity. This issue can have detrimental effects on soil quality. Furthermore, these compounds can exert negative influences on the chemical properties of the soil^12^. Petroleum hydrocarbons are causing obstruction of pore spaces between soil particles, elevating soil temperature and diminishing the presence of beneficial microorganisms. Additionally, these pollutants impede the absorption of nutrients by plants, ultimately resulting in plant mortality^13^. In recent years, innovative methods for bioremediation of soil pollutants have been researched and applied, yielding favorable results from these studies^14–16^. Several chemical and physical methods exist to address HMs pollution in soil, such as incineration and solvent-based extraction. However, these approaches are often deemed less effective due to their high costs and incomplete removal of pollutants. Currently, there is a growing emphasis on biological methods for the removal of contaminants from soil^17^.

Phytoremediation is affordable and biological process that utilizes plants to eliminate and break down pollutants, including HMs, petroleum compounds, and various other toxic organic substances, from contaminated soil^18^. This technology relies on the integration of plant activity and the microbial community to nourish, transfer, deactivate, and immobilize soil pollutants. Phytoremediation can address the removal of pollutants through five distinct forms: phytostabilization, phytoextraction, phytodegradation, rhizofiltration, and phytovolatilization. In addition to garnering public acceptance, this method represents a novel and sustainable approach that is both suitable and cost-effective, particularly for developing countries^19,20^.

In oil-rich areas, it is crucial to identify plant species capable of remediating contaminated soils. Native plant species adapted to polluted area environments can offer practical phytoextraction potential, particularly plants that tolerate drought, salinity, and contamination. In this study, based on field observations and previous research, the *A. camelorum* plant was selected for the phytoremediation of TPHs and HMs. This plant, belonging to the *Fabaceae* family, is renowned for its deep-rooted nature and resilience to cold and drought conditions^21^, it flourishes particularly well in the southern regions of Iran. In recent years, numerous studies on phytoremediation have been conducted to eliminate pollutants from the soil. Nevertheless, most phytoremediation research has focused on controlled greenhouse studies utilizing artificial pollutant spiking rather than field assessments under natural conditions. Additionally, few studies have characterized the kinetics of pollutant removal to better gauge required remediation timescales. The aim of this study was to investigate these research gaps and assess the phytoremediation capacity of *A. camelorum* for TPHs and HMs in oil-contaminated soils in a field-scale level. Therefore, we pursued three main objectives: (i) the *A. camelorum's* impact on this pollutants removal, (ii) its effect on soil microbial activity, and (iii) investigation of kinetic modeling for the removal rate of pollutants. The findings from this field-realistic phytoremediation assessment, using pollution-resistant native species, can offer practical strategies for mitigating the risks of oil pollution.

## Materials and methods

### Study area

**Figure 1:**
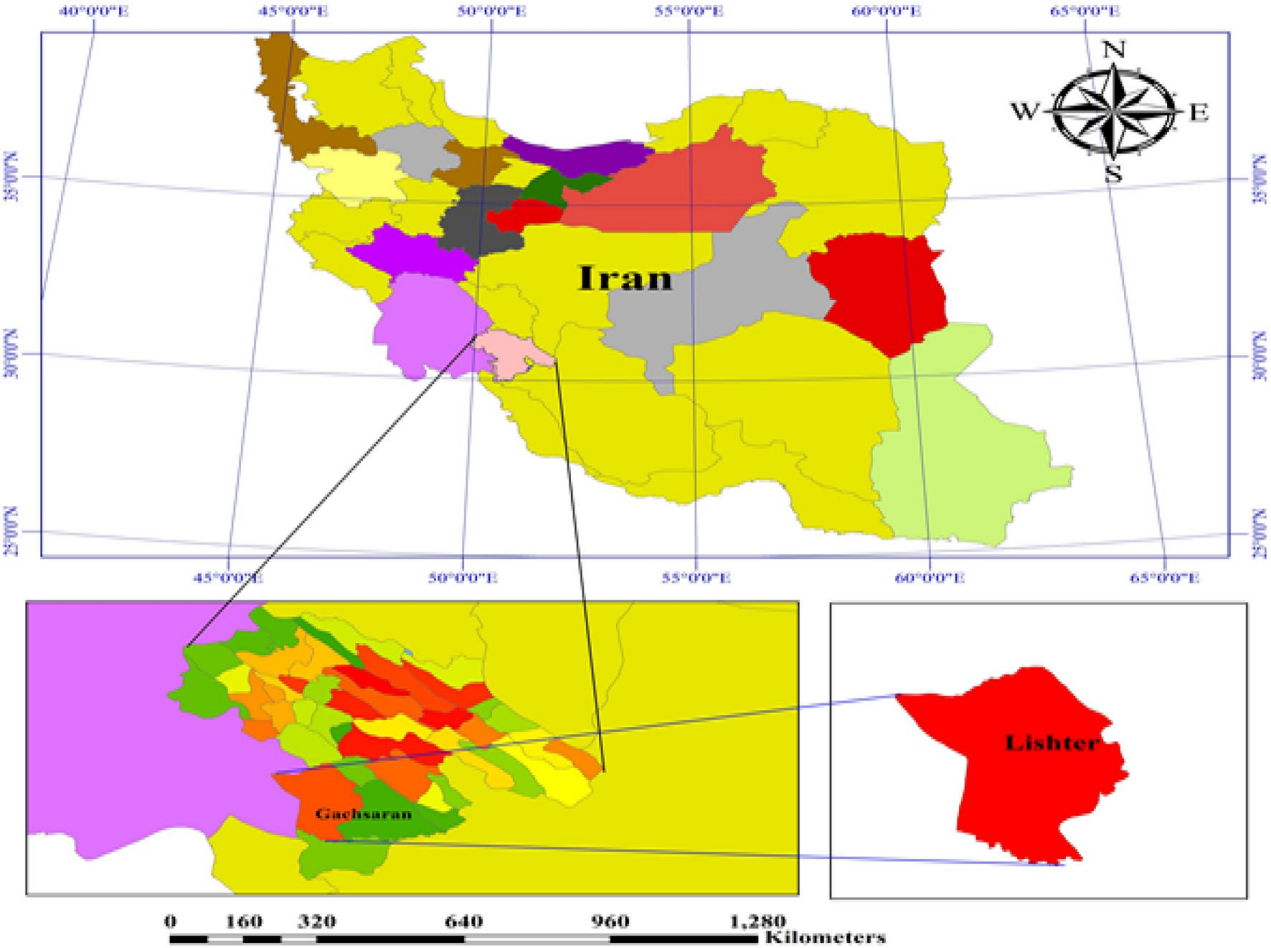
Location of the study area.

The present study was conducted in the southwest country of Iran and the oil-rich region of Lishter, located in Gachsaran oil field and in close proximity to oil wells with geographical coordinates of 30°28′06.7"N 50°30′54.9"E. The Gachsaran oil Field (Fig. 1) stands as one of Iran's oldest and most renowned oil-industrial regions. Boasting reserves of approximately 52 billion barrels of crude oil, it ranks as the country's second-largest oil field, following the Ahvaz oil field. Positioned at an average elevation of 720 m above sea level, this field experiences a tropical climate. The surrounding region is home to a population exceeding 120,000 individuals. With an average annual temperature of 22.5 °C and an annual precipitation of 441 mm, freezing temperatures occur only two days a year. The vegetation cover in this area consists of both annual and perennial herbaceous species, as well as shrubby plants^22^. Given the daily extraction of crude oil, coupled with refining and oil transportation operations, the soils in the vicinity of this area are susceptible to significant oil pollution. Additionally, the substantial population residing nearby, combined with agricultural activities and crop harvesting from lands in proximity, poses potential risks to human health.

### Soil preparation and plant cultivation

The soil under investigation was collected from 12 points in an oil-rich area at a depth of 0–20 cm. To ensure soil homogeneity, a sieve with a mesh size of number 10 (equivalent to 2 mm openings) was employed. Following mixing, the soil was irrigated for two weeks until it reached field capacity moisture, and turning every three days. The research site was prepared and excavation, and its floor has been covered with a geomembrane. Subsequently, contaminated soils, with a height of 50 cm, were transferred to the research site and leveled and compacted into plots (100 × 100 cm). The seeds used in this study were sourced from the Research Institute of Forests and Rangelands of Iran. The plant seeds were germinated and nurtured in a greenhouse environment (a nursery) using uncontaminated soil. After one month, seedlings of similar sizes and visual characteristics (height of 14 cm) were harvested and transferred to the designated soil plots and watering and plant care were carried out. Given the resilience of this plant and its low water requirements for growth, as well as to align with the actual environmental conditions of the region, watering was performed only until the seedlings initiated growth. Three replicates were conducted for each plot, and control plots (without plant cultivation) were also considered.^23^.

### Phytoremediation experiment

This study spanned a duration of six months. At the end of each month (every 30 days), soil samples were collected from all plots (containing plants and controls), and measurements were taken for HMs, TPHs, and microbial respiration of the soil^23^. Additionally, soil bacterial enumeration was performed at the beginning of the study, after 90 days, and after 180 days.

#### Soil sampling

Soil sampling was conducted using sterile spatulas at 10 points within each plot at a depth of 0–10 cm. Stones and foreign materials were removed, and after mixing and homogenizing the collected soil, 100 g of soil were transferred to the laboratory for analysis. All stages of sampling and sample preparation were carried out according to standard methods^24^. The samples were stored at 4 °C until the final analysis.

#### Measurement and analysis of TPHs

The Agilent GC‒MS Model 5975 and the QuEChERS (Quick, Easy, Cheap, Effective, Rugged, and Safe) technique were employed for the measurement of TPHs. The effectiveness of this method in extracting hydrocarbons and organic compounds from soil, plants, foods, and animal samples has been reported^25^. To initiate the analysis, 10 g of the dried sample were placed in a 50 mL centrifuge tube and shaken for 1 min with 3 mL of water, followed by an another minute of shaking with 15 mL of dichloromethane. A mixture of salts (4 g MgSO_4_, 1 g sodium chloride (NaCl), 0.5 g disodium hydrogen citrate sesquihydrate, 1 g trisodium citrate dehydrate) was added to the sample and shaken for another minute. The sample was centrifuged at 4000 rpm for 8 min and then transferred to a clean centrifuge tube. Following this, the sample was filtered using a 45 μm nylon filter disk. Subsequently, 300 μL of the sample was injected into the GC‒MS instrument with 700 μL of dichloromethane for analysis. The temperature was initially set at 50 °C for 1 min, then increased to 120 °C at a rate of 25 °C/min, further to 160 °C at a rate of 10 °C/min, and then to 240 °C at a rate of 6 °C/min. Finally, it reached 315 °C at a rate of 2 °C/min and held for 10 min. High-purity helium carrier gas was employed with a flow rate of 1.2 mL/min^26^.

#### Measurement and analysis of HMs

The analysis of HMs (Pb, Cr, Ni, and Cd) was conducted following standard measurement methods, utilizing an OPTIMA 2100 DV ICP‒OES^27^. Initially, samples were dried in an oven at 70 °C, sieved through a 2 mm mesh, and prepared for extraction. Subsequently, 15 mL of 4 N nitric acid was added to 2 g of dried and sieved soil, which was then placed in an oven at 60 °C for 20 h. The samples were subsequently brought to volume in a 50 cc clean flask and distilled twice with water. In the next step, the samples were filtered through cellulose acetate filter paper (23 μm) to prepare them for analysis using an ICP‒OES device.

#### Contaminant removal and degradation

The pollution removal efficiency was calculated as a percentage based on Eq. 1.

1 $${\text{R }}\left( \% \right) \, = \, \left( {{\text{C}}_{0} - {\text{C}}_{{\text{t}}} } \right)/{\text{C}}_{0} \times {1}00$$

where R represents the efficiency of pollutant removal (%), C_o_ is the initial pollutant concentration (mg/L), and C_t_ is the pollutant concentration at the end of the study (mg/L)^28^.

### Soil microbial activity

This study did not involve any microbial inoculation. The investigation concentrated exclusively on assessing the plant's impact on the natural soil microbial population's activity, considering soil respiration and analyzing the count of heterotrophic bacteria.

To determine the total number of heterotrophic bacteria in the soil, 1 g of soil was poured into a test tube containing 9 mL of sodium chloride solution (9000 mg/L). The obtained mixture was diluted to create dilutions ranging from 10^−1^ to 10^−8^. Then, the diluted solutions were transferred to nutrient-rich agar medium. The plates were placed in an incubator at a temperature of 28 °C for 48 h. Subsequently, the formed colonies were counted. The microbial population according to Eq. 2 is expressed in colony-forming units (CFU) per g of soil^29^. A colony counter device was used for counting bacterial colonies.

2 $${\text{Number of CFU}}/{\text{mL}} = \, \left( {\text{Number of CFU}} \right)/({\text{Volume plated }}({\text{mL}}) \times {\text{Total dilution used}})$$

Microbial respiration was monitored over a six-month period at monthly intervals. The measurement of CO_2_ produced by microbial respiration was carried out using the residual sodium hydroxide (NaOH) titration method^30^. Initially, 25 g of each sample was placed in dedicated microbial respiration containers, and distilled water, up to 70% of the agricultural capacity, was added to each sample. In each container, an experimental tube containing 10 mL of 0.5 N NaOH was placed, and the container lids were tightly closed. Subsequently, the containers were placed in an incubator at a temperature of 25 °C. At the designated time intervals, the experimental tubes were extracted from within the microbial respiration containers, and their contents were transferred into Erlenmeyer flasks. Subsequently, 10 mL of 10% barium chloride and a few drops of phenolphthalein indicator were introduced to each sample, and the contents of the Erlenmeyer flasks were titrated with 0.25 N sulfuric acid. Finally, the quantity of carbon produced as CO_2_ was computed based on the amount of acid generated, according to Eq. 3.

3 $${\text{C}}_{{\text{t}}} = \, \left[ {\left( {{\text{B}} - {\text{S}}} \right).{\text{ N}}.{\text{ E}}.{1}000} \right]/{\text{W}}$$

C_t_ is the amount of carbon released due to microbial respiration (mg/kg), B is the volume of acid consumed for control samples (mL), S is the volume of acid consumed by the sample (mL), N is the normality of acid consumed, E is the carbon equivalent weight, W is the weight of dried oven soil (g), and 1000 is the conversion factor from soil to kg.

### Kinetic rate modeling

Kinetic modeling is a key factor in understanding the biological removal process, measuring the rate of environmental biodegradation, and developing effective remedies for crude oil-contaminated environments. Information related to the kinetics of soil bioremediation is of paramount importance, as it determines the concentration of residual pollution at any given time and enables the calculation of the time required for soil remediation. The pollutant removal rate using the kinetic equation based on the first-order rate^31^, as described in Eq. 4, was examined.

4 $${\text{ln C}}_{{\text{t}}} = {\text{lnC}}_{0} {-}k{\text{t}}$$

where C_t_ represents the concentration of a parameter at time t (mg/L), C_0_ represents the initial concentration of the parameter at time t (mg/L), *k* is the first-order rate constant (day^-1^), and t represents time (days).

The half-life (t_1/2_) is the time needed for the pollutant concentration to decrease by half of its initial concentration. The value is as observed in Eq. 5.

5 $${\text{t}}_{{{1}/{2}}} = {\text{ ln 2}}/k = \, 0.{6932}/k$$

### Statistical analysis

The data were analyzed using SPSS 22 (IBM). Descriptive statistics were employed to calculate the mean and standard deviation. The Independent Samples Test was used for comparing TPHs, HMs, microbial respiration, and microbial counts before and after plant cultivation, compared with the control group. Temporal changes in the mentioned variables were assessed using Repeated Measure test. The significance level of (*p* < 0.05) was considered.

## Results and discussion

### Physical and chemical properties of the soil

Given the significant role of the physical and chemical properties of the studied soil in the phytoremediation process^32^, soil samples were analyzed before planting. Some of these properties are listed in Table 1. Additionally, the results of measuring TPHs and HMs (Pb, Cr, Ni, and Cd) are presented in this table.

**Table 1:** Physical and chemical characteristics of raw soil.

Parameter	Measuring method	Results ± SD
Organic carbon (%co)	Walkley–Block	2.06 ± 0.62
Nitrogen (%N)	Kjeldal	0.21 ± 0.05
Phosphorus (mg/kg)	Olsen	32.84 ± 4.2
Alkalinity (pH)	Digital pH meter	7.11 ± 1.12
Electrical conductivity (ds/m)	EC meter	1.89 ± 0.28
TPHs (mg/kg)	GC‒MS	861.5 ± 22.3
Pb (mg/kg)	ICP‒OES	6.55 ± 0.78
Cr (mg/kg)	ICP‒OES	16 ± 1.06
Ni (mg/kg)	ICP‒OES	46.5 ± 2.12
Cd (mg/kg)	ICP‒OES	0.33 ± 0.02
Soil texture		
Clay	Hydrometry	10.05 ± 2.38
Sand	Hydrometry	62.3 ± 4.51
Silt	Hydrometry	27.65 ± 2.86

### The TPHs removal from soil

**Figure 2:**
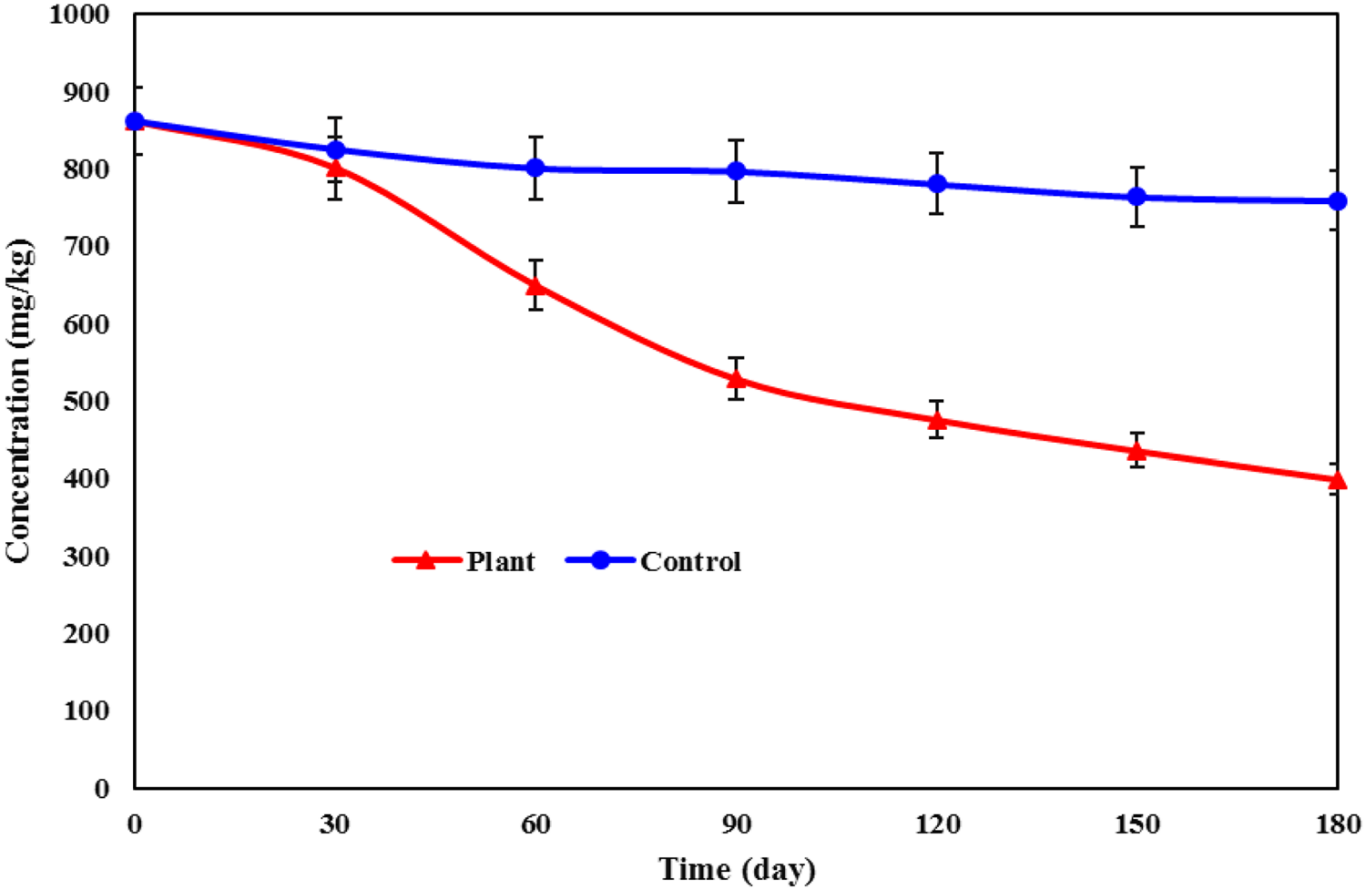
TPHs removal rate in soil with *A. camelorum.*

The study results demonstrated that the average concentration of TPHs in the soil contaminated with petroleum compounds significantly decreased (*p* < 0.05) in the plots where *A. camelorum* was present compared to the control group (53.6 ± 2.8% versus 11.87 ± 2.2%). Over a 180-day period, the initial concentration of TPHs in the soil decreased from 861.5 ± 22.3 in plots with the plant to 399.2 ± 16.5, while the concentration in the control plates reached 759.2 ± 19 (Fig. 2). Considering the significant difference in meaning between the effect of plant presence and the control plots, the reduction in petroleum hydrocarbon concentration can be attributed to the effective role of the *A. camelorum* in removing the petroleum pollutants. *Alhagi camelorum* has the characteristic of hyperaccumulation of soil hydrocarbons and can be effective not only in absorbing various elements but also in transferring these elements from the roots to the aerial parts^33^. The research conducted by Aisien et al. showed that phytoremediation stands out as one of the most efficient and cost-effective techniques for diminishing hydrocarbons in the soil, and offering an environmentally friendly approach. The authors also underscored the imperative to encourage a greater inclination toward phytoremediation for the removal of soil pollutants^34^.

Numerous studies offer substantial evidence supporting the effective removal of hydrocarbon pollutants by native plants. For example, phytoremediation employing *maize* plant can achieve a removal rate of 70%, while *sorghum* and *barley* demonstrate removal percentages ranging from 52 to 64%, *Lolium perenne* exhibits a removal efficiency of 45.6%, and *Iris dichotoma* removes approximately 30.79% of the TPHs present in the soil^35–38^. On the flip side, several studies have indicated a direct correlation between the enhancement of petroleum hydrocarbon degradation and the microbial population in contaminated soil under plant cultivation when compared to uncultivated soil. This phenomenon occurs because plant roots create a conducive environment for microbial activity and growth, fostering a larger microbial population, particularly in the root zone. Consequently, this leads to the degradation and breakdown of petroleum compounds^32^.

Based on the data obtained from the control plots (Fig. 2), it is observed that around 12% of the petroleum hydrocarbon pollutants have been removed, likely due to processes like evaporation and oxidation. When a soil experiences an oil spill, petroleum hydrocarbons undergo distinct physicochemical processes in the environment, including evaporation and photochemical oxidation, leading to alterations in the composition of the oil. However, the primary and crucial process among these is biodegradation^39^. Although resistant plants can degrade petroleum hydrocarbons and separate them from the soil environment, various factors, including pollutant behavior and concentration, plant handling, oxygen, nutrients, moisture, soil acidity and alkalinity, and other environmental factors, can affect their efficiency^40^. Another contributing factor to the reduction of oil hydrocarbons in the soil column is leaching induced by irrigation water. This process leads to the mobilization and transportation of oil compounds to lower soil layers^41^. Throughout the study and over time, a progressively higher percentage of oil hydrocarbons was eliminated. After approximately 30 days, the rate of hydrocarbon decomposition intensified, and then gradually decreasing until the end of the study, and their removal proceeded at a slower pace. The findings of the Gavrilescu et al*.* study align with these results, indicating that plants exhibit their highest capacity for absorbing pollutants during the initial stages of growth. Nevertheless, as time progresses, their ability to absorb pollutants gradually diminishes, resulting in a decline in the percentage of pollutants they can uptake^42^. The most significant TPHs removal percentage occurred within the time range of 30–60 days (18.93%), whereas the lowest efficiency was noted in both the initial time range (0–30 days) and the final time range (150–180 days), registering percentages of 7.02% and 8.37%, respectively. The results of the Ekperusi et al*.* study support these findings, revealing that the optimal removal efficiency for hydrocarbons was observed in the time range of 15–30 days (16.78%), while the lowest removal percentage (2.15%) was recorded in the time span of 105–120 days^43^. Some studies conducted under greenhouse conditions have demonstrated more favorable outcomes in the removal of TPHs^44,46^. It is probable that the improved results in TPHs removal observed in greenhouse studies are attributed to the controlled environmental conditions. In greenhouses, plants tend to experience better growth, with their roots becoming more extensive, ultimately contributing to a more effective removal of hydrocarbons.

### The HMs removal from soil

**Figure 3:**
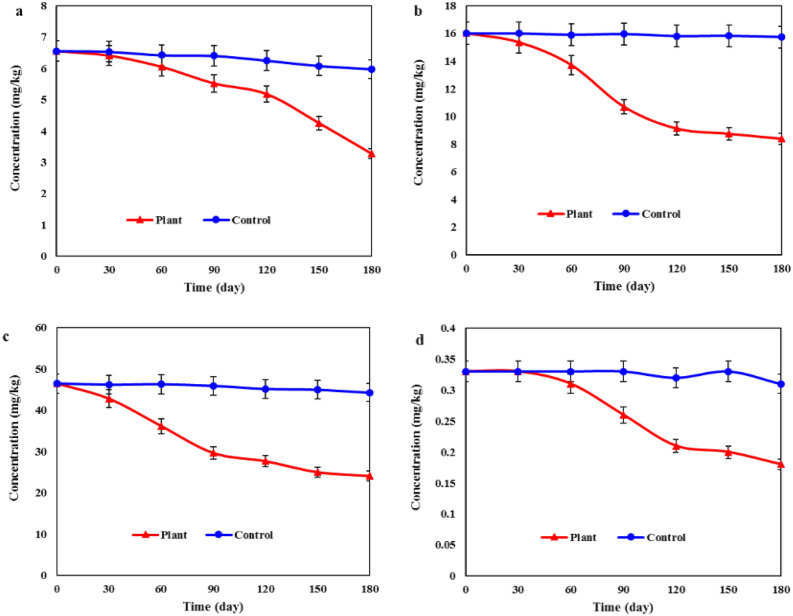
HMs removal rate in soil with *A. camelorum*: (**a**) Pb, (**b**) Cr, (**c**) Ni, (**d**) Cd.

Figure 3 depicts the trend of changes in HMs in soil over six months. At the end of the study period, the average removal of Pb, Cr, Ni and Cd in plots containing the *A. camelorum* was 50 ± 2.1, 47.6 ± 2.5, 48.1 ± 1.6, and 45.4 ± 3.5%, respectively. In the control plots, the average removal was 8.7 ± 1.2, 1.7 ± 0.6, 4.7 ± 0.9 and 6 ± 1.4%, respectively. The results indicate that the presence of plants has had a significant effect on the separation of HMs from the soil.

The reduction in concentration of all four metals analyzed in plots containing plants was found to be statistically significant (P < 0.05) when compared to control plots. Prior studies have indicated that the biological accumulation factor of *A. camelorum* for Pb and zinc was higher than one, suggesting that this plant holds potential for the phytoremediation of HMs-contaminated soils^47^. A study conducted in 2020 demonstrated that phytoremediation is an appropriate method for reducing HMs in the soil. After a 7-month period, the average removal rates for HMs of Cu, As, Pb, Hg, V, Zn, Cd, and Ni were 23%, 39%, 60%, 54%, 27%, 36%, 44%, and 38%, respectively^48^. Other studies have identified the *A. camelorum* as an efficient and effective hyperaccumulator plant for phytoremediation of HMs^33,49^. Hyperaccumulator plants have a high capacity for accumulating HMs and can accumulate 100 to 1000 times more HMs than ordinary plants^50^. The bioremediation of HMs can be facilitated by plants, microorganisms, or a synergistic combination of both.

In the course of the phytoremediation process, HMs accumulate in plant tissues and may undergo transformation into other compounds through oxidation. The oxidation of HMs can reduce their toxicity by converting them into less harmful compounds that are more readily volatilized, water-soluble, and can be effectively removed through leaching^51^. In this study, the highest percentage of HMs removal was observed for Pb. Some plants have the capacity to accumulate Pb in their tissues at levels surpassing 50 mg/g of dry weight of the plant^52^. In a phytoremediation study conducted under laboratory conditions, plants were able to remove over 90% of the Pb present in the soil. The roots of plants demonstrated a notable ability to extract a high percentage of Pb from the soil^53^. The greater accumulation of HMs in the roots, compared to the aerial parts, suggests the plant's ability to withstand elevated concentrations of metals. This issue is particularly important, especially in the case of Pb, because it has been determined that this element mainly accumulates in the roots. Plants that can store Pb in their roots can be a suitable option for phytoremediation^54,55^.

The average reduction in Cr concentration in plots containing the plant compared to the control was also significant. *A. camelorum* has demonstrated the ability to reduce Cr concentrations by approximately 48% in the soil. The plant's roots can absorb hexavalent Cr ions from the soil. Then, through the use of specific enzymes and chemicals, hexavalent Cr is converted into trivalent Cr^56^. Hexavalent Cr is toxic to all plants and animals, posing a significant carcinogenic risk. It demonstrates high solubility and accessibility in both water and soil^57^. Cr is predominantly stored in the roots of plants and usually has higher concentrations in the roots than in the stems and leaves^58^.

After Pb, the highest percentage of HMs removal from the soil was specifically associated with Ni. This metal is an essential micronutrient for plants, and even a small amount of it can be highly beneficial, enhancing plant growth^59^. Nevertheless, at elevated concentrations, it can compete with other metals in absorption and prevent the uptake of essential metal ions. This situation can be problematic as it may interfere with the overall nutrient balance and adversely impact the health of plants^60^. Previous studies have indicated that employing various plant species for phytoremediation can serve as a suitable method for the separation of Ni from soil^61,62^. For example, research has demonstrated that the biological saturation factor of the *Stipagrostis plumosa* plant is greater than one, indicating its effectiveness as a suitable choice for Ni phytoremediation^33^. In another study, it was found that over 4 months, *Jatropha curcas* and *Pongamia pinnata* plants removed 82–86% and 90–93% of Ni, respectively^63^.

The phytoremediation efficiency of *A. camelorum* in removing Cd was approximately 45% (Fig. 3). In plots containing the plant, there were significant changes in Cd levels compared to the control, indicating the beneficial effect of the plant in reducing soil Cd. The mobility of Cd in the soil prompts the plant to extract it from the soil^64^. For this reason, phytoremediation is an appropriate method for removing Cd from the soil^65^. Studies on phytoremediation with *Virola surinamensis*, *Miscanthus giganteus*, *oats* and *white mustard* obtained favorable results in removing Cd^66–68^, all of which confirm the results of the present study.

### Heterotrophic bacterial population

The population of heterotrophic bacteria in the soil was measured at the beginning of the study, after 90 days and after 180 days, and the results are presented in Table 2.

**Table 2:** Population of heterotrophic bacteria in soil (CFU/g).

Time (day)	Control Plots	Plots with plants
0	1.94 × 10^6^	1.94 × 10^6^
90	3.88 × 10^6^	5.11 × 10^6^
180	6.08 × 10^6^	8.51 × 10^6^

The number of bacteria in plots containing *A. camelorum* was exceeded that in control plots, and a significant difference was observed at the 5% level. The presence of plant species in the soil is associated with an augmentation in microorganisms^69^. The amount of remaining TPHs in the soil is inversely proportional to the level of microorganisms present in the soil, and the greatest reduction in pollutants occurs in the rhizosphere due to the increase in microorganisms in this area^32^. The rhizosphere, by increasing the number of decomposing bacteria, increasing the secretion of chemical compounds, and stimulating plants, destroys oil pollutants^25^. Although many microorganisms are capable of degrading the crude oil present in the soil, the biodegradation capabilities of bacteria have been increasingly recognized. Bacteria that can degrade petroleum products include Pseudomonas, Aeromonas, Moraxella, Flavobacter, Corinobacteria, Nocardia, Acinetobacteria, Mycobacteria, Arthrobacter, and Cyanobacteria^70^.

The efficiency of the phytoremediation process relies on the presence and activity of the microbial community associated with the plant^71^. The population of microorganisms in the soil within the rhizosphere of plants is several times greater than that in soil without the presence of plant roots^72^. Plants' roots also release organic compounds such as amino acids, sugars, and carbohydrates, stimulate the growth and activity of microorganisms^73^. For example, it has been reported that the activity of *grass* and *alfalfa* plants increases the population and capability of hydrocarbon-degrading bacteria, including microbacteria and Pseudomonas^74^. Several studies have been conducted on bacterial growth in soil in the presence of plant roots, all of which confirm that over time and with the expansion of plant roots, bacterial growth has also increased^75,76^.

### Microbial respiration

**Figure 4:**
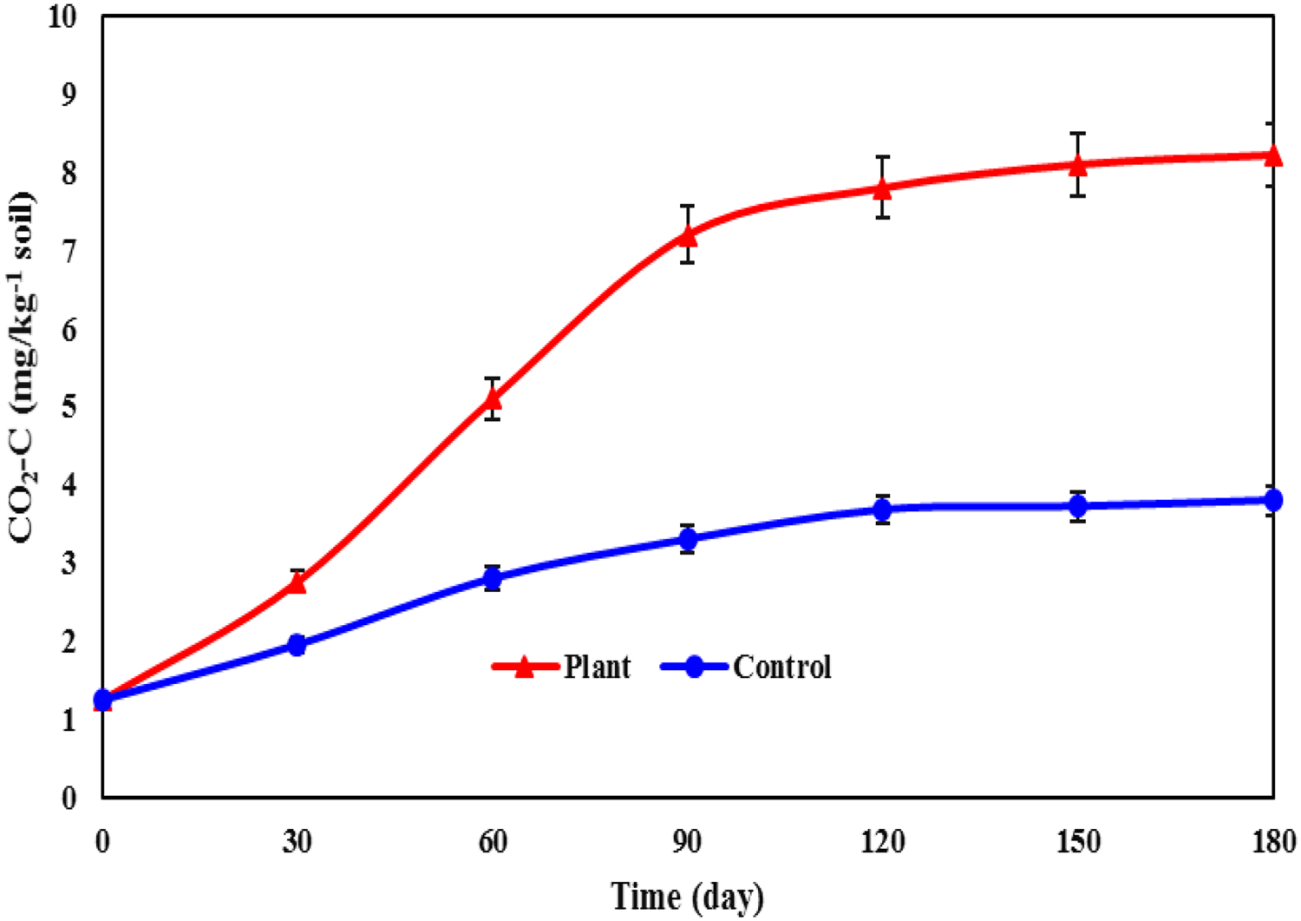
The effect of *A. camlorum* on soil microbial respiration (mg/kg^−1^).

The comparison of carbon production as CO_2_ from microbial respiration in soils containing plants and the control showed a significant difference. In plots with plants, the quantity of carbon produced exhibited substantial growth over time, as depicted in Fig. 4.

A comparison of the average data in Fig. 4 demonstrates the impact of plants on soil microbial respiration. In contaminated soils and in the presence of plants, the level of microbial respiration increases because both pollutants and plants stimulate the microbial population and intensify the activity of some microorganisms, which is probably due to the role of pollutants as substrates for the soil microbial community and resident microorganisms^77^. Numerous studies have established a direct correlation between microbial respiration and the level of pollutant degradation^78,79^. Plants with strong roots systems can accelerate the activity of microorganisms and the decomposition of pollutants by facilitating the transfer of oxygen and nutrients in the vicinity of their roots^80^. In this study, the role of plants in increasing microbial activity and consequently the degradation of pollutants was also confirmed. As we approached the end of the study, the upward slope of microbial respiration decreased, which could be due to a decrease in hydrocarbons over time. A study conducted in 2015 supported this observation, indicating a significant rise in carbon production as CO_2_ in hydrocarbon-contaminated soils compared to non-contaminated soils^81^. Additionally, Polyak et al. concluded that the peak of soil microbial respiration occurred at the initiation of the experiment when hydrocarbon concentrations were at their highest. The high concentration of hydrocarbons stimulates microbial activity and enhances microbial respiration^82^.

### Kinetic rate of pollutants removal in soil

The outcomes derived from the application of the first-order kinetic equation to model the removal rate of all five pollutants (TPHs, Pb, Cr, Ni, and Cd) by the *A. camelorum* plant are outlined in Table 3. The results stemming from the remediation of these pollutants, taking into account the rate constant (*k*), half-life (t_1/2_), and goodness of fit (R^2^) in the soil treated with *A. camelorum*, demonstrated a commendable alignment with the first-order kinetic rate model.

**Table 3:** First-order kinetics of the rate model for pollutants in soil.

Parameters	Treatments	First-order rate	K(day^-1^)	T_1/2_ (days)	R^2^
TPHs	Control	Y = −0.00026x–0.03728	0.00026	2665.4	0.1457
	Plant	Y = −0.00457x–0.00166	0.00457	151.6	0.9721
Pb	Control	Y = 0.00012x–0.02202	0.00012	5775	0.0237
	Plant	Y = −0.00364x + 0.09565	0.00364	190.4	0.8798
Cr	Control	Y = −0.00010x + 0.00138	0.00010	6930	0.8677
	Plant	Y = −0.00414x + 0.02738	0.00414	167.4	0.9446
Ni	Control	Y = −0.00023x + 0.00501	0.00023	3013	0.7857
	Plant	Y = −0.00393x–0.01316	0.00393	176.3	0.9645
Cd	Control	Y = −0.00026x + 0.01007	0.00026	2665.4	0.4713
	Plant	Y = −0.00382x + 0.07818	0.00382	181.4	0.9455

The R^2^ values for TPHs, Pb, Cr, Ni and Cd pollutants were obtained with coefficients of 0.9721, 0.8798, 0.9446, 0.9645 and 0.9455, respectively, indicating that the removal of all five pollutants follows the first-order kinetic model. The phytoremediation of TPHs by *Lemna paucicostata* over 120 days, yielding an R2 coefficient of 0.938 and a rate constant of 0.0325, exhibited strong conformity with the first-order kinetic rate model^43^. Singh et al. reported that in terms of HMs removal, the first-order model provided better results with a coefficient of determination (R^2^ > 0.82) and rate constant (k > 0.023 mgl^−1^d^−1^)^83^. Several other studies have demonstrated that the decrease in oil pollutants adheres to a first-order kinetic rate model during the phytoremediation process^84,85^. The values of the removal rate constant (k) and half-life (t_1/2_) are presented in Table 3. Elevated values of k indicate a faster pollutant removal rate and a more substantial reduction in its half-life. The findings revealed that k values for all five pollutants (TPHs, Pb, Cr, Ni, and Cd) were higher in the presence of plants compared to the control plots, and the half-life of all pollutants decreased in the presence of plants.

### Limitations

Cultivating and maintaining plants in open-field is a complex process that demands precise control and adequate safeguarding. Furthermore, eliminating pollutants from soils with lower contamination levels poses greater challenges and yields less efficiency. It is worth noting that, as artificial pollutants were not employed in this study, the removal efficiency was comparatively lower compared to certain similar studies.

### Practical implications

The findings of this research provide important recommendations for the phytoremediation management and control of oil pollutants in soil. One of the key factors for successful phytoremediation is the utilization of native plants. Additionally, native plants should have the ability to grow in polluted areas and harsh environmental conditions. Therefore, a phytoremediation experiment can be successful when the conditions of the studied plants are examined. Furthermore, since the findings indicate that the presence of plants enhances soil microbial activity, adding plants and microorganisms to oil-contaminated soils can leverage the synergistic capability of plant–microbe interactions for pollutant removal.

## Conclusion

This study investigated the impact of *A. camelorum* on HMs and TPHs in soil under field conditions. Over the 180-day experiment, *A. camelorum* significantly reduced TPHs and HMs in contaminated soils compared to unplanted controls. *A. camelorum* also improved key soil health indicators, increasing heterotrophic bacteria and soil microbial respiration. Furthermore, the first-order kinetic models demonstrated a substantial fit, with compatibility exceeding 80%, for the removal of the studied pollutants by this plant. Although this study achieved favorable results from the phytoremediation over a six-month period, it should be noted that *A. camlorum* is a perennial plant, and the efficiency of pollutant removal may vary over the plant's lifespan in subsequent years (due to changes in stems and roots). Therefore, it is suggested to pay attention to this aspect in future studies and investigate the pollutant removal efficiency in the later years of the plant's lifespan.

## Data Availability

Data supporting the findings of this study are available from the corresponding author.

## Abbreviations

ICP‒OES: Inductively coupled plasma-optical emission spectroscopy
GC‒MS: Gas chromatography‒mass spectrometry
HMs: Heavy metals
TPHs: Total petroleum hydrocarbons
Cd: Cadmium
Cr: Chromium
Pb: Lead
Ni: Nickel
